# The Non-ureogenic Stinging Catfish, *Heteropneustes fossilis*, Actively Excretes Ammonia With the Help of Na^+^/K^+^-ATPase When Exposed to Environmental Ammonia

**DOI:** 10.3389/fphys.2019.01615

**Published:** 2020-01-22

**Authors:** Shit F. Chew, Stephanie Z. L. Tan, Sabrina C. Y. Ip, Caryn Z. Pang, Kum C. Hiong, Yuen K. Ip

**Affiliations:** ^1^Natural Sciences and Science Education, National Institute of Education, Nanyang Technological University, Singapore, Singapore; ^2^Department of Biological Sciences, National University of Singapore, Singapore, Singapore

**Keywords:** air-breathing fishes, carbamoyl phosphate synthetase, nitrogen metabolism, ornithine-urea cycle, Singhi catfish, ureogenesis

## Abstract

The stinging catfish, *Heteropneustes fossilis*, can tolerate high concentrations of environmental ammonia. Previously, it was regarded as ureogenic, having a functional ornithine-urea cycle (OUC) that could be up-regulated during ammonia-loading. However, contradictory results indicated that increased urea synthesis and switching to ureotelism could not explain its high ammonia tolerance. Hence, we re-examined the effects of exposure to 30 mmol l^–1^ NH_4_Cl on its ammonia and urea excretion rates, and its tissue ammonia and urea concentrations. Our results confirmed that *H. fossilis* did not increase urea excretion or accumulation during 6 days of ammonia exposure, and lacked detectable carbamoyl phosphate synthetase I or III activity in its liver. However, we discovered that it could actively excrete ammonia during exposure to 8 mmol l^–1^ NH_4_Cl. As active ammonia excretion is known to involve Na^+^/K^+^-ATPase (Nka) indirectly in several ammonia-tolerant fishes, we also cloned various *nka*α*-subunit* isoforms from the gills of *H. fossilis*, and determined the effects of ammonia exposure on their branchial transcripts levels and protein abundances. Results obtained revealed the presence of five *nka*α*-subunit* isoforms, with *nka*α*1b* having the highest transcript level. Exposure to 30 mmol l^–1^ NH_4_Cl led to significant increases in the transcript levels of *nka*α*1b* (on day 6) and *nka*α*1c1* (on day 1 and 3) as compared with the control. In addition, the protein abundances of Nkaα1c1, Nkaα1c2, and total NKAα increased significantly on day 6. Therefore, the high environmental ammonia tolerance of *H. fossilis* is attributable partly to its ability to actively excrete ammonia with the aid of Nka.

## Introduction

In fishes, proteins ingested would be first catabolized to amino acids and then further broken down through deamination or transamination with the eventual production of ammonia. Although the production of ammonia can occur in many tissues (Walton and Cowey, 1977), the liver is the main organ where it is produced (Pequin and Serfaty, 1963). Most teleost fishes are ammonotelic, keeping body ammonia levels low by excreting excess ammonia mainly as NH_3_ (>50% of the total nitrogenous wastes) across the gills (Wright et al., 1993). In aqueous solution, ammonia exists as molecular NH_3_ and cationic NH_4_^+^, based on the reaction NH_3_ + H_3_O^+^ ⇔ NH_4_^+^ + H_2_O with a pK of 9.0–9.5. Hence, acidic environmental conditions augment ammonia excretion, as NH_3_ diffusing across the branchial epithelium is converted into NH_4_^+^ and trapped in the external environment. By contrast, an increase in the pH of the environment would impede the diffusion of NH_3_ into the external medium leading to the accumulation of endogenous ammonia in the body. When fishes emerge from water, there could be a reduction in the excretion of ammonia due to a lack of water to flush the gills. Furthermore, ammonia concentrations in the ambient water could increase due to the decomposition of organic matters or the addition of fertilizers, and impede ammonia excretion in fishes.

As aquatic hypoxia is a frequent event in tropical waters, air-breathing is a common occurrence (Graham, 1997). With the development of air-breathing abilities, some tropical fishes can leave water and make short excursions on land, while some even burrow into semi-solid mud during drought. When confronted with terrestrial conditions or low levels of environmental (exogenous) ammonia, fishes may have difficulties in excreting ammonia that is endogenously produced. However, when confronted with high environmental ammonia concentrations that result in a reversed P_NH__3_ gradient, fishes would have to detoxify not only endogenous ammonia but also exogenous ammonia that could have penetrated into the body (ammonia-loading). Under all these adverse environmental conditions, ammonia accumulation in the fish’s body would occur, but ammonia is toxic (see Ip et al., 2001a; Chew and Ip, 2014 for reviews) and fishes are generally very susceptible to elevated tissue ammonia levels. Therefore, many tropical fish species have evolved mechanisms to avoid ammonia accumulation and to ameliorate ammonia toxicity during terrestrial or ammonia exposure (see Ip et al., 2001a, 2004a,b; Chew et al., 2006; Ip and Chew, 2010, 2018; Chew and Ip, 2014, 2017 for reviews).

The stinging (or Singhi) catfish, *Heteropneustes fossilis*, belonging to Family Heteropneustidae (air sac catfishes), can be found in Pakistan, India, Bangladesh, Myanmar, and Thailand (Burgess, 1989; Munshi and Hughes, 1992). Its main habitats include ponds, ditches, swamps, marshes, and muddy rivers (Munshi and Choudhary, 1994). It can survive long periods of emersion and tolerate moderately high concentrations of environmental ammonia (Saha and Ratha, 1990, 1994; Saha et al., 2001). It had been reported that *H. fossilis* was ureogenic and contained a functional ornithine-urea cycle (OUC) that could be up-regulated during ammonia-loading (Saha and Ratha, 1987, 1989, 1994, 1998; Saha et al., 1997). However, as pointed out by Graham (1997), those reports also comprised intrinsically contradictory evidence, indicating that ureogenesis and shifting from ammonotelism (excreting > 50% of the total waste-N as ammonia) to ureotelism (excreting > 50% of the total waste-N as urea) could not be the “major” contributor to its high ammonia tolerance.

Saha and Ratha (1990) reported that *H. fossilis* absorbed ammonia from the external environment when exposed to 25, 50, or 75 mmol l^–1^ NH_4_Cl for 28 days. There was an increase in urea excretion by 1.5- to 2-fold between day 10 and day 12, which remained high throughout. Thus, they concluded that “prolonged hyper-ammonia stress induced the shift from ammonotelism to ureotelism in *H. fossilis*.” However, their results did not corroborate such a conclusion. This is because the rate of ammonia−N excretion reported in the control fish fasted for 14 days was 7.82 μmol 48 h^–1^ g^–1^ (Table 1 in Saha and Ratha, 1990) and a fish exposed to 75 mmol l^–1^ NH_4_Cl for 14 days absorbed 56.37 μmol ammonia−N 48 h^–1^ g^–1^. If one assumes that ammonia−N excretion was totally impeded in a medium containing 75 mmol l^–1^ NH_4_Cl, the total ammonia−N accrued would be 7.82 + 56.37 = ∼64 μmol 48 h^–1^ g^–1^. Yet, the increase in the rate of urea excretion after exposure to 75 mmol l^–1^ NH_4_Cl was reported to be only 2.86 (urea excretion rate in 75 mmol l^–1^ NH_4_Cl) − 0.96 (urea excretion rate in water) = 1.90 μmol (or 3.80 μmol urea−N) 48 h^–1^ g^–1^ (Saha and Ratha, 1990, Table 2). Hence, the increase in urea excretion represented ∼5.93%, which is only a very small fraction, of the total accumulated ammonia−N (∼64 μmol ammonia−N 48 h^–1^ g^–1^). Alternatively, it can be interpreted that *H. fossilis* could have actively excreted a certain amount of ammonia during environmental ammonia exposure; however, this possibility had not been examined. Another enigma was that the rate of ammonia absorption from the external environment increased with increasing concentrations of external NH_4_Cl (Table 1 in Saha and Ratha, 1990), but the rate of urea excretion was relatively constant during exposure to different concentrations of external NH_4_Cl (Table 2 in Saha and Ratha, 1990). If indeed increased urea synthesis and shifting from ammonotelism to ureotelism were the “major” strategies adopted by *H. fossilis* to survive ammonia exposure, an increase in urea excretion rate should have occurred with an increase in the concentration of environmental ammonia. These discrepancies led Graham (1997) to state that “paradoxically, some of the species reported to have the OUC enzymes are not ureotelic; urea, in fact, amounts to a quite a small percentage of the total nitrogen excreted by *Heteropneustes* (even in 75 mmol l^–1^ NH_4_Cl).”

At present, the roles of ureogenesis and ureotelism in defending ammonia toxicity in *H. fossilis* exposed to environmental ammonia remain controversial. Therefore, this study was undertaken to re-examine the effects of exposure to 30 mmol l^–1^ NH_4_Cl on the rates of ammonia and urea excretion, and the tissue ammonia and urea concentrations, in *H. fossilis*. As results obtained contradicted information in the literature (Saha and Ratha, 1987, 1989, 1990), we decided to re-examine the presence of carbamoyl phosphate synthetase (CPS) III activity in its liver using an established radiometric method (Anderson et al., 1970; Anderson, 1980; Anderson and Walsh, 1995; Chew et al., 2003b, 2004; Tam et al., 2003; Ip et al., 2012a). This is because all the reports that indicated the possible presence of CPS III in the liver of *H. fossilis* were based on a coupled-enzyme colorimetric assay (Saha and Ratha, 1987; Saha et al., 1997). Subsequently, we obtained results that denoted a lack of detectable CPS III activity in the liver of *H. fossilis*, demanding an alternative explanation for its high ammonia tolerance. Thus, we made an effort to examine whether *H. fossilis* could actively excrete ammonia during ammonia exposure (8 mmol l^–1^ NH_4_Cl). This is because active ammonia excretion has been reported previously for the highly ammonia-tolerant giant mudskipper, *Periophthalmodon schlosseri* (Peng et al., 1998; Randall et al., 1999; Ip et al., 2005; Chew et al., 2007), climbing perch, *Anabas testudineus* (Tay et al., 2006), and African sharptooth catfish, *Clarias gariepinus* (Ip et al., 2004d). In the case of *A. testudineus* (Ip et al., 2012b, c; Loong et al., 2012) and *P. schlosseri* (Chew et al., 2014, 2015), Na^+^/K^+^-ATPase (Nka) is known to take part in a multi-component mechanism involved in active ammonia excretion. Therefore, we also cloned and sequenced isoforms of *nka*α*-subunits* from the gills of *H. fossilis*, and determined the effects of ammonia exposure on their branchial transcript levels and protein abundances, which could serve as an indicator of the ability of *H. fossilis* to actively excrete ammonia.

## Materials and Methods

### Catfish and Rearing Condition

*Heteropneustes fossilis* (approximately 20–60 g) were imported from India through a local fish importer (Qian Hu Fish Farm, Singapore). Its identity was confirmed by the very small dorsal fin that lacked a leading spine and was located in the anterior third of body, as well as the two tubular air sacs that extended from the gill cavity to the caudal peduncle (Burgess, 1989). The fish (without sex differentiation) were acclimated in plastic tanks containing dechlorinated fresh water at a temperature of 25–26°C and under a photoperiod of 12 h illumination and 12 h darkness using artificial light. The fish were fed frozen bloodworms and the water in the tanks was changed daily for a week. Feeding of the fish was halted 2 days before experimentation. Approval of protocol (ARFSBS/NIE-A-0311) for fish maintenance and experimentation was granted by the Nanyang Technological University Institutional Animal Care and Use Committee.

### Effects of Exposure to 30 mmol l^–1^ NH_4_Cl on Urea Excretion Rate

Fish (20–30 g) were exposed individually in a tank containing 20 vol (volume to the mass of the fish) of either fresh water (five individuals in the control group) or fresh water containing 30 mmol l^–1^ NH_4_Cl (five individuals in the experimental group; pH 7.0) for 6 days. The water in the tanks was replaced daily. On day 1, 3, or 6 (after 24 h of water replacement), 2 ml of water was sampled, acidified with 0.2 ml of H_2_SO_4_, kept at 4°C, and used for the analysis of urea within a week. The colorimetric method by Jow et al. (1999) was used for the determination of urea. The excretion rate was expressed as μmol urea−N g^–1^ day^–1^.

### Effects of Exposure to 8 mmol l^–1^ NH_4_Cl on Ammonia and Urea Excretion Rate

Fish (20–25 g) were immersed in a small plastic container containing six volumes (volume to the mass of the fish) of fresh water (five individuals) or fresh water with 8 mmol l^–1^ NH_4_Cl (five individuals) (pH 7.0). Temperature was kept at 25–26°C. A water sample (1 ml) was taken immediately as the 0 h sample. After 24, 48, and 72 h, 1 ml of water was again sampled. The water sampled was acidified as mentioned above. The water was kept at 4°C and used for both ammonia and urea analyses within a week. A parallel set of containers without fish was set up and water was sampled for ammonia analysis to ensure that the ammonia concentration was not altered significantly by bacterial action. The colorimetric method by Anderson and Little (1986) was used for ammonia analysis. The rate of ammonia excretion was expressed as μmol ammonia−N g^–1^ day^–1^.

### Effects of Exposure to 30 mmol l^–1^ NH_4_Cl on Ammonia and Urea Concentrations in Tissues

Fish (25–40 g) were exposed individually in a tank containing 30 mmol l^–1^ NH_4_Cl (pH 7.0) for 1 day (five individuals), 3 days (five individuals), or 6 days (15 individuals) with daily changes of NH_4_Cl solution. Fish kept individually in fresh water (15 individuals) served as controls. At the respective time interval, fish were sacrificed by immersing in 0.1% phenoxyethanol and then applying a blow to the head. Gills, muscle, and liver were dissected, frozen in liquid nitrogen, and kept in a −80°C freezer. A separate group of fish (30–60 g) used for blood collection was exposed to either fresh water (10 individuals) or fresh water containing 30 mmol l^–1^ NH_4_Cl for 6 days (10 individuals). The fish was anesthetized and heparinized capillary tubes were used to collect the blood from the caudal peduncle. The plasma obtained after centrifugation of the blood at 4000 × *g* for 10 min at 4°C were deproteinized in two volumes of ice-cold 6% trichloroacetic acid. The clear supernatant obtained after centrifugation of the deproteinized samples at 10,000 × *g* for 15 min at 4°C was used for analyses of ammonia and urea concentrations.

The muscle and liver tissues collected were first ground to a powder in liquid nitrogen, before weighing and homogenizing in five volumes (w/v) of ice-cold 6% trichloroacetic acid using an Ultra-Turrax disperser (Ika-Werke, Staufen, Germany) set to 24,000 r/min for 20 s thrice. The clear supernatant obtained after centrifugation of the homogenate as mentioned above was used for analyses of ammonia and urea concentrations.

The method by Bergmeyer and Beutler (1985) was used for the determination of ammonia concentrations in the tissue samples while the urea concentrations were analyzed as mentioned above.

### Determination of CPS Activities in the Liver

The extraction procedure was carried out on liver samples using the method of Ip et al. (2012a). Approximately 300 mg of liver was homogenized in five volumes (w/v) of ice-cold buffer containing 50 mmol l^–1^ Hepes (pH 7.6), 50 mmol l^–1^ KCl, 0.5 mmol l^–1^ EDTA, 0.5 mmol l^–1^ phenylmethanesulfonyl fluoride (PMSF), and 1 mmol l^–1^ dithiothreitol as mentioned above. The homogenate was sonicated three times at 110 W, 20 kHz using a Misonix sonicater (Farmingdale, NY, United States) for 20 s each with two 10 s off-intervals. The sonicated homogenate was then centrifuged as mentioned above, and the supernatant collected was desalted using the Econo-Pac 10DG desalting column (Bio-Rad Laboratories, Hercules, CA, United States) equilibrated already with extraction buffer without EDTA and PMSF. The eluent collected was used for CPS assay. The protein concentrations of all samples (before and after elution) were determined using the Bio-Rad Protein assay dye (Bradford, 1976) in order to obtain the dilution factor to be used in the subsequent calculation for CPS activities. CPS activity was determined using the radiometric method described by Anderson and Walsh (1995). Briefly, the reaction mixture consisted of 0.05 mol l^–1^ Hepes (pH 7.5), 0.05 mol l^–1^ KCl, 0.024 mol l^–1^ MgCl_2_, 0.2 mmol l^–1^ EDTA, 0.4 mmol l^–1^ dithiothreitol, 0.02 mol l^–1^ ATP, and 5 mmol l^–1^ [^14^C]bicarbonate. The substrates tested were 10 mmol l^–1^ glutamine (for CPS III assay) or 100 mmol l^–1^ NH_4_Cl (for CPS I assay) or glutamine + NH_4_Cl (for CPS III + CPS I assay). The inclusion of 0.5 mmol l^–1^ *N*-acetylglutamate in the assay medium was used to activate CPS I or III activity, while 1 mmol l^–1^ UTP was added to inhibit the activity of CPS II. A Wallac 1414 liquid scintillation counter (Wallac Oy, Turku, Finland) was used for radioactivity measurement. CPS activity was expressed as μmol [^14^C]urea formed min^–1^ g^–1^.

### Extraction of Total RNA From Gills and cDNA Synthesis

The extraction and purification of total RNA from gill samples and the synthesis of cDNA from the purified RNA were performed following the method described in Chew et al. (2014). Briefly, Tri Reagent^TM^ (Sigma–Aldrich Co., St. Louis, MO, United States) was used for the extraction while RNeasy Plus Mini Kit (Qiagen GmbH, Hilden, Germany) was used for the purification. The quantification of the RNA obtained was performed with a NanoDrop ND-1000 spectrophotometer (Nanodrop Technologies Inc., Wilmington, DE, United States) and the integrity of the RNA was verified using gel electrophoresis. The purified RNA was converted to cDNA with oligo (dT)_18_ primers using the RevertAid^TM^ First Strand cDNA synthesis kit (Thermo Fisher Scientific Inc., Waltham, MA, United States).

### Cloning and Sequencing of *nkaα-subunit* Isoforms

A set of conserved primers (forward 5′−3′ sequence: CACTTCATCCACATCATCAC; reverse 5′−3′ sequence: ATGGCAGGGAACCATGTC) was designed to obtain the partial nucleotide sequences of *nkaα-subunit* as described by Chew et al. (2014). Briefly, the polymerase chain reaction (PCR) was performed in a 9902 Veriti 96-well thermal cycler (Applied Biosystems, Carlsbad, CA, United States) using DreamTaq^TM^ polymerase (Thermo Fisher Scientific Inc.). The amplification protocol was as follows: an initial denaturation for 3 min at 94°C, followed by 35 cycles of 94°C for 30 s, 55°C for 30 s, 72°C for 2 min, and one cycle of final elongation at 72°C for 10 min. The products obtained were separated by gel electrophoresis and the bands of the estimated amplicon size were extracted and purified using the Promega Wizard SV gel and PCR cleanup system (Promega Corporation, Madison, WI, United States). These purified products were cloned into pGEM^®^-T Easy vector (Promega Corporation) following the method used by Chew et al. (2014). The sequencing was carried out using the BigDye^®^ Terminator v3.1 Cycle Sequencing Kit (Applied Biosystems) and the 3130XL Genetic Analyzer (Applied Biosystems). The BioEdit version 7.1.11 (Hall, 1999) software was used to assemble and analyze the sequences obtained. After checking and verifying that the sequences obtained were *nkaα-subunit*, the full nucleotide sequence of all the isoforms of *nkaα-subunit* was obtained through Rapid amplification of cDNA ends (RACE-PCR) using RACE-PCR primers (Supplementary Table S1) and the SMARTer^TM^ RACE kit (Clontech Laboratories, Mountain View, CA, United States). The cDNA sequences of the isoforms of *nkaα-subunit* were identified and deposited into GenBank, and the accession numbers are Nkaα1b (MH427004), Nkaα1c1 (MH427006), Nkaα1c2 (MH427005), Nkaα2 (MH427007), and Nkaα3 (MH427008).

### Characterization and Phylogenetic Analysis of the Deduced Nkaα Amino Acid Sequences

The amino acid sequences of the various isoforms of Nkaα were deduced using the ExPASy Proteomic server^1^ (Gasteiger et al., 2003). The identities of the Nkaα isoforms from *H. fossilis* were confirmed by aligning and comparing with Nkaα isoforms sequences from selected teleosts retrieved from the Genbank database to generate a sequence identity matrix. The potential phosphorylation sites and transmembrane domains of the Nkaα isoforms were predicted using NetPhos 2.0 and MEMSAT3 and MEMSAT-SVM provided by PSIPRED protein structure prediction server^2^, respectively (McGuffin et al., 2000).

Selected protein sequences of Nkaα isoforms of teleosts and *Saccoglossus kowalevskii* NKAα1 (as outgroup) retrieved from GenBank database were used to construct the phenogram. Phylogenetic analysis was conducted using Phylip (Felsentein, 1989) with bootstrapping values taken from 1000 replicates via the neighbor-joining method. The phenogram constructed further confirmed the identities of the Nkaα isoforms.

### Determination of *nkaα-*transcript Levels by Quantitative Real-Time PCR (qPCR)

The cDNA used for quantification of the transcripts levels of various isoforms of *nkaα-subunits* was synthesized using random hexamer primers provided in the cDNA synthesis kit mentioned above. Absolute quantification of the transcripts levels of *nkaα-subunit* isoforms was performed as described in Chew et al. (2015). Briefly, the qPCR reaction was accomplished in a volume of 25 μl consisting of 2 × KAPA SYBR^®^ FAST Master Mix ABI Prism^®^ (Kapa Biosystems, Wilmington, MA, United States), 0.3 μmol l^–1^ forward and reverse qPCR primers (Supplementary Table S1), and 1 ng of cDNA or appropriate standards. Each PCR was carried out in triplicates in a StepOnePlus^TM^ Real-Time PCR System (Applied Biosystems). The amplification protocol consisted of an initial denaturation at 95°C for 3 min followed by 35 cycles at 95°C for 30 s, 60°C for 30 s, 72°C for 30 s, and a final cycle of elongation at 72°C for 10 min. The threshold cycle (*C*_*t*_) value for each well was collected and a melt curve analysis was conducted to confirm that a single product was obtained. In addition, agarose gel electrophoresis on the product was performed to verify that only one band was present. The amplification efficiencies of the primers for *nkaα1b, nkaα1c1*, *nkaα1c2, nkaα2*, and *nkaα3* obtained were 84.5, 84.5, 86.2, 89.3, and 92.2%, respectively. The quantity of *nkaα-subunit* isoforms transcripts in a sample was determined using the standard curve prepared for each *nkaα-subunit* isoform and expressed as copies of transcripts per ng total RNA.

### Antibodies

Two isoform-specific anti-Nkaα antibody against Nkaα1c1 or Nkaα1c2 of *H. fossilis* were custom-made by GenScript (Piscataway, NJ, United States). They were: (1) a rabbit anti-Nkaα1c1 polyclonal antibody against amino acid residues 19–32 (GNKKSKSKGKKDKD) of Nkaα1c1 and (2) a mouse anti-Nkaα1c2 polyclonal antibody against amino acid residues 469–478 (GGMREKYPKV) of Nkaα1c2. The anti-NKAα antibody (α5), which is known to react comprehensively with multi-Nkaα isoforms (developed by Douglas M. Farmbrough, Johns Hopkins University, Baltimore, MD, United States) was purchased from the Developmental Studies Hybridoma Bank (Department of Biological Sciences, The University of Iowa, Iowa City, IA, United States) while the anti-β-actin antibody (MA5-11869) was purchased from Thermo Fisher Scientific Inc.

### Measurement of NKAα Protein Abundance by Western Blotting

The gill samples collected were homogenized thrice in five volumes (w/v) of extraction buffer as described in Chew et al. (2015) in a Precellys homogenizer (Bertin Instruments, Montigny-le-Bretonneux, France) set at 6000 r/min for 20 s. After homogenization, the homogenate was centrifuged and the protein concentration was quantified in the supernatant according to Chew et al. (2015). Protein samples were diluted with Laemmli’s buffer (Laemmli, 1970) so that 20 μg of protein samples was loaded onto the gel (comprising 8% acrylamide for the resolving gel and 4% acrylamide for the stacking gel) in the Mini-PROTEAN^®^ Tetra Vertical Electrophoresis Cell apparatus (Bio-Rad Laboratories). Subsequently, the separated proteins were transferred onto nitrocellulose membranes using a mini Transblot Cell (Bio-Rad Laboratories). The separated proteins were detected using the Pierce Fast Western Blot kit, SuperSignal^®^ West Pico Substrate (Thermo Fisher Scientific Inc.). The membranes were incubated with anti-Nkaα1c1 antibody (1:5000 dilution), or anti-Nkaα1c2 antibody (1:3000 dilution) or anti-α5 (1:8000 dilution). To detect the reference protein, the membranes were incubated with pan-anti-actin antibody (1:15,000 dilution) for 1 h at 25°C. Subsequently, the membranes were incubated with either anti-rabbit or anti-mouse horseradish peroxidase-conjugated secondary antibody for 15 min at 25°C. The imaging of the blots was performed using the ChemiDoc Imager (Bio-Rad Laboratories). The intensity of the bands were quantified using the ImageJ software (version 1.40, NIH), calibrated with a 37-step reflection scanner scale (1” x 8”; Stouffer #R3705-1C). The protein abundance was expressed as relative protein abundance of Nka per μg protein normalized with pan-actin. A peptide competition assay was performed on the anti-Nkaα1c1 and anti-Nkaα1c2 antibodies to confirm the identity of the band of interest for each antibody. The anti-Nkaα1c1 (1 μg) and anti-Nkaα1c2 (3.33 μg) antibodies were pre-incubated with their corresponding immunizing peptide (4 and 33.33 μg, respectively) from Genscript at 25°C for 1 h before performing the peptide competition assay.

### Statistical Analysis

The SPSS software version 25 (IBM Corp., Armonk, NY, United States) was used for all statistical analysis. Measured or generated data were presented as means ± standard errors of means (SEM). All data were evaluated for equality of variance using the Levene’s test. For testing the difference between the freshwater (control) group and the ammonia-exposed group, student’s *t*-test was applied while for testing among the freshwater (control) group and/or among the ammonia-exposed group at different time interval, one-way analysis of variance was applied. When a significant difference was detected for the means among the groups, multiple comparisons of the means using Dunnett’s T3 were performed when the variance was unequal while Tukey’s test was applied when the variance was equal. The means were considered as statistically different with *P* < 0.05.

## Results

### Effects of 30 mmol l^–1^ NH_4_Cl on Urea Excretion Rate

**FIGURE 1:**
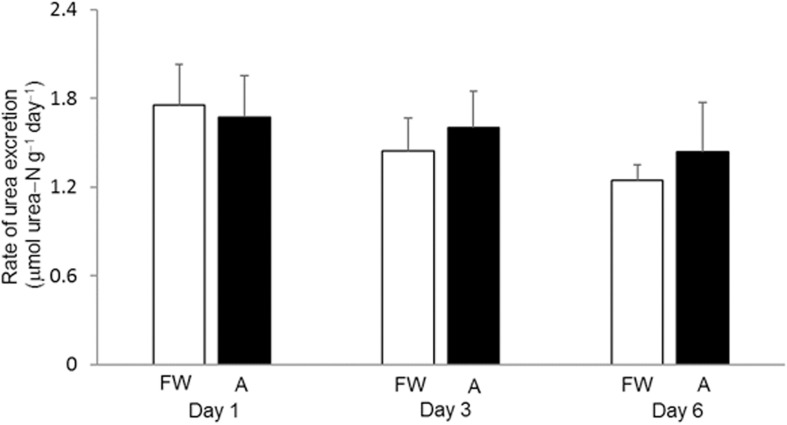
Effects of exposure to 30 mmol l^–1^ NH_4_Cl on the rate of urea excretion in *Heteropneustes fossilis*. Rate of urea excretion (μmol urea−N g^–1^ day^–1^) in *H. fossilis* kept in fresh water (FW; open bar) or exposed to 30 mmol l^–1^ NH_4_Cl (A; closed bar) for 1, 3, or 6 days. Values are mean + SEM (*N* = 5).

The daily urea excretion rate of *H. fossilis* kept in fresh water was relatively low, ranging from 1.24 to 1.76 μmol urea−N g^–1^, and it remained statistically unchanged during 6 days of exposure to 30 mmol l^–1^ NH_4_Cl (Figure 1). It was technically not possible to determine the rate of ammonia excretion due to the presence of such a high concentration of ammonia (30 mmol l^–1^ NH_4_Cl) initially in the water.

### Effects of 30 mmol l^–1^ NH_4_Cl on Tissue Ammonia and Urea Concentrations

**FIGURE 2:**
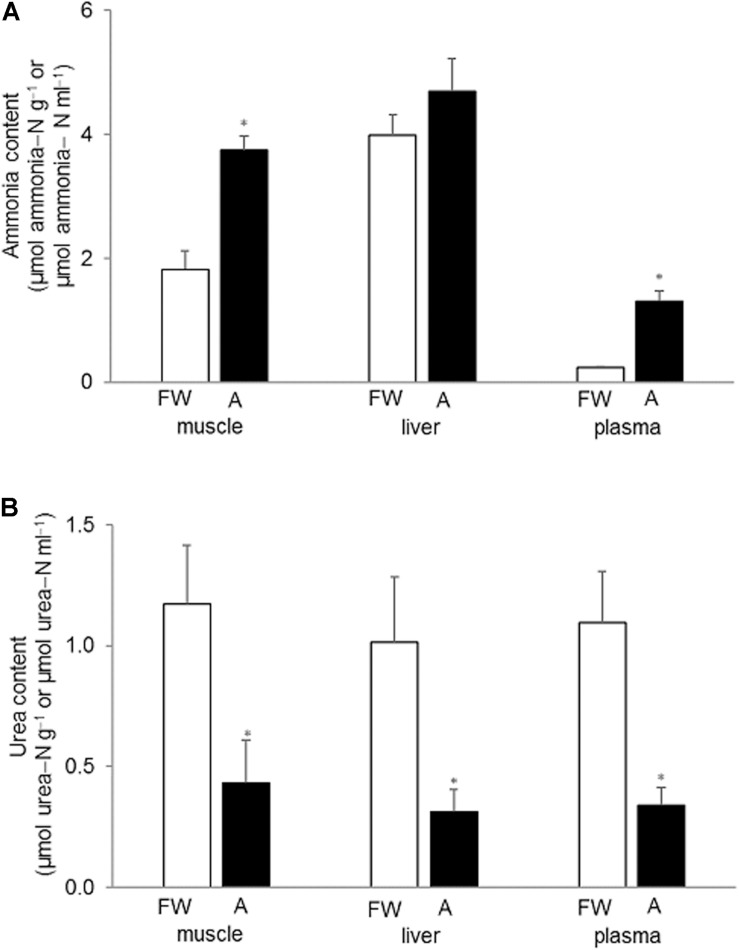
Effects of exposure to 30 mmol l^–1^ NH_4_Cl on ammonia and urea concentrations in the tissues of *Heteropneustes fossilis*. Concentrations (μmol ammonia−N or urea−N g^–1^ tissue or μmol ammonia−N or urea−N ml^–1^ plasma) of **(A)** ammonia and **(B)** urea in the muscle liver and plasma of *H. fossilis* kept in fresh water (FW; open bar) or exposed to 30 mmol l^–1^ NH_4_Cl (A; closed bar) for 6 days. Values are mean + SEM (*N* = 5). *Significantly different from the corresponding tissue or plasma of the FW fish, *P* < 0.05.

Generally, the muscle and liver of *H. fossilis* contained much more ammonia than urea (Figures 2A,B). Exposure to 30 mmol l^–1^ NH_4_Cl for 6 days led to significant increases in ammonia contents in the muscle (∼twofold) and plasma (∼fivefold) but not the liver (Figure 2A). Surprisingly, the urea content in the muscle, liver, and plasma decreased significantly (by ∼65%) after exposure to 30 mmol l^–1^ NH_4_Cl for 6 days (Figure 2B).

### Effects of 8 mmol l^–1^ NH_4_Cl on Rates of Ammonia and Urea Excretion

**FIGURE 3:**
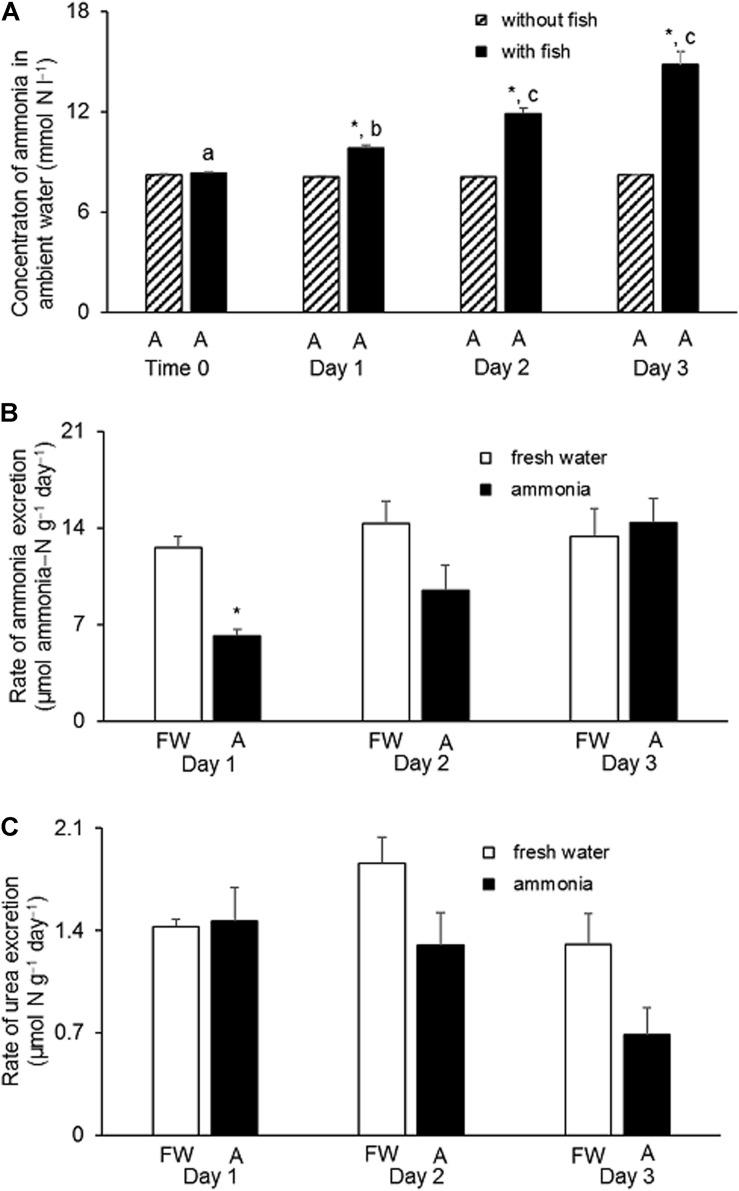
Effects of exposure to 8 mmol l^–1^ NH_4_Cl on rates of ammonia and urea excretion in *Heteropneustes fossilis*. **(A)** Concentrations of ammonia in the external medium containing 8 mmol l^–1^ NH_4_Cl at time 0 or on day 1, 2, or 3 without (shaded bar) or with (closed bar) the incubation of *H. fossilis*. Rates (μmol ammonia−N or urea−N g^–1^ day^–1^) of **(B)** ammonia and **(C)** urea excretion in *H. fossilis* exposed to the same external medium of fresh water (FW; open bar) or fresh water containing 8 mmol l^–1^ NH_4_Cl (A; closed bar) on day 1, 2, or 3. Values are mean + SEM (*N* = 5). ^∗^Significantly different from that of fish kept in fresh water, *P* < 0.05. Means not sharing the same letter are significantly different (*P* < 0.05).

When *H. fossilis* was exposed to water containing 8 mmol l^–1^ NH_4_Cl, there was a progressive and significant increase in the ammonia concentration, from 8 to 15 mmol ammonia−N l^–1^, in the ambient water during a 3-day period (Figure 3A). This indicated that *H. fossilis* was able to continuously excrete endogenously and metabolically produced ammonia during exposure to 8 mmol l^–1^ NH_4_Cl. For the control fish kept in fresh water for 3 days, the daily rate of ammonia−N excretion ranged between 12.7 and 14.7 μmol g^–1^ day^–1^ (Figure 3B). In comparison, fish exposed to 8 mmol l^–1^ NH_4_Cl had a positive ammonia−N excretion rate ranging from 6.2 to 9.8 μmol g^–1^ day^–1^. Although the rate of ammonia excretion was significantly lower than the freshwater control on day 1 of ammonia exposure, it recovered to the control level on day 2 and day 3, indicating the possible up-regulation of certain mechanisms of active ammonia excretion (Figure 3B). By contrast, the daily rate of urea−N excretion in control fish kept in fresh water was very low (ranging from 1.2 to 2.0 μmol g^–1^ day^–1^, and it remained unchanged during 3 days of exposure to 8 mmol l^–1^ NH_4_Cl (ranged from 0.68 to 1.46 μmol g^–1^ day^–1^) (Figure 3C).

### CPS III and CPS I Activities Are Undetectable in the Liver

Using the radiometric method employed in this study, no CPS I activity was detected in the liver of *H. fossilis* when NH_4_^+^ was used as the substrate (Table 1). Although a very low activity was detected when glutamine was used as the substrate, it could not be CPS III activity as it was refractory to *N*-acetyl-glutamate activation (Table 1). The results of CPS activities from the mouse, *Mus musculus*, and the stingray, *Taeniura lymma*, presented in Table 1 were extracted from Ip et al. (2012a), which were obtained using the same method in the same laboratory.

**TABLE 1:** Activities (μmol [^14^C]urea min^–1^ g^–1^ liver; *N* = 4) of carbamoyl phosphate synthetase (CPS/Cps), determined in the presence of various substrates and effectors, from the liver of *Heteropneustes fossilis*, *Mus musculus* (mouse; with CPS I), and *Taeniura lymma* (stingray; with CPS III).

Substrate and effectors	CPS/Cps activity (μmol min^–1^ g^–1^ liver)		
	*H. fossilis*	*M. musculus* (CPS I)	*T. lymma* (Cps III)
NH_4_Cl	n.d.	n.d.	0.008 ± 0.007
NH_4_Cl + *N*AG	n.d.	4.01 ± 0.43	0.09 ± 0.02
NH_4_Cl + *N*AG + UTP	n.d.	3.94 ± 0.25	0.06 ± 0.02
Glutamine	0.009 ± 0.005	n.d.	0.23 ± 0.04
Glutamine + *N*AG	0.008 ± 0.004	n.d.	0.63 ± 0.10
Glutamine + *N*GA + UTP	n.d.	n.d.	0.60 ± 0.11
NH_4_Cl + Glutamine + *N*AG	0.006 ± 0.004	4.08 ± 0.46	0.59 ± 0.09

*Activities from *M. musculus* and *T. lymma* were taken from Ip et al. (2012a). *N*AG, *N*-acetyl-L-glutamate; UTP, uridine triphosphate; n.d., not-detectable (detection limit = 0.001 μmol min^–1^ g^–1^ wet mass).*

### Nucleotide Sequences, Translated Amino Acid Sequences, and Phylogenetic Analysis of *nka*α/Nkaα in the Gills

Five complete coding cDNA sequences of *nka*α*-subunit* were obtained from the gills of *H. fossilis*. They were deposited into GenBank with accession numbers presented in Table 2. The cDNA sequences ranged from 3063 to 3078 bp, and the translated amino acids sequences ranged from 1021 to 1026 residues. The molecular mass of these *nka*α*-subunit* isoforms ranged from 112.6 to 113.2 kDa (Table 2).

**TABLE 2:** cDNA length (bp), amino acid length, and estimated molecular mass (kDa) of *Na^+^/K^+^-ATPase* (*nka*) *α-subunit* isoforms.

Gene	Accession No.	cDNA length (bp)	Amino acid length	Estimated molecular mass (kDa)
*nkaα1b*	MH427004	3066	1022	112.9
*nkaα1c1*	MH427006	3078	1026	113.2
*nkaα1c2*	MH427005	3072	1024	112.8
*nkaα2*	MH427007	3063	1021	112.6
*nkaα3*	MH427008	3066	1022	112.6

**FIGURE 4:**
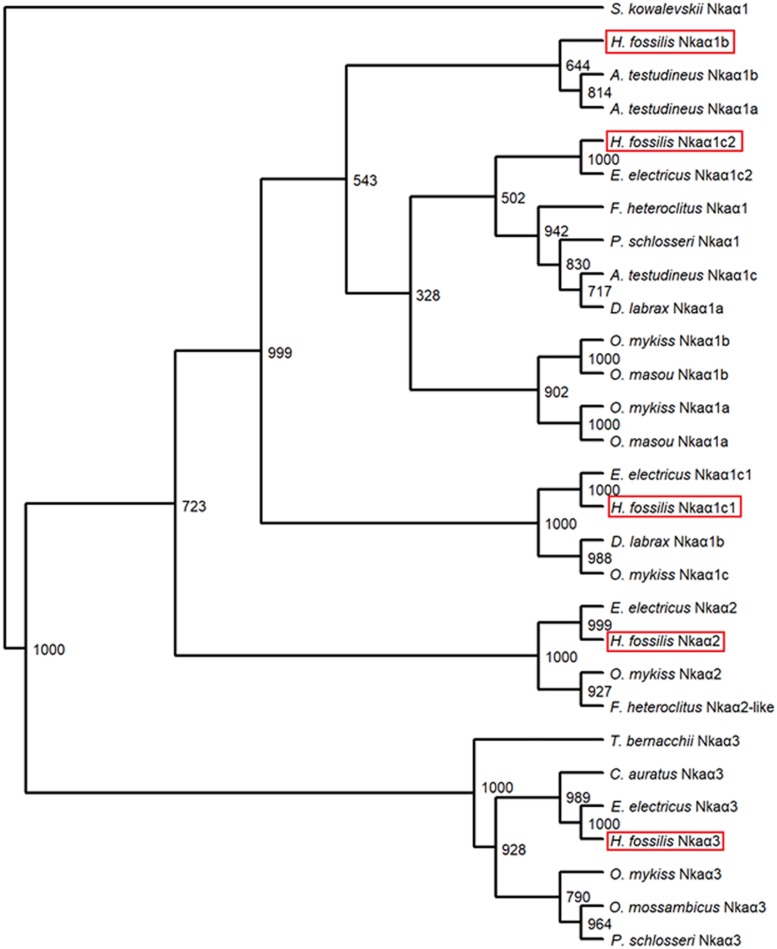
A phenogramic analysis of Na^+^/K^+^-ATPase α (Nkaα) isoforms from the gills of *Heteropneustes fossilis*. A neighbor-joining tree was constructed to illustrate the relationship between Nkaα isoforms from the gills of *H. fossilis* and Nkaα/NKAα of selected teleost species. The number at each node represents the bootstrap value obtained from 1000 replicates. NKAα1 of *Saccoglossus kowalevskii* was selected as an outgroup. The accession numbers of the amino acid sequences of Nkaα-subunit isoforms obtained from Genbank database were as follows: *Anabas testudineus* Nkaα1a (JN180940.1), *A. testudineus* Nkaα1b (JN180941.1), *A. testudineus* Nkaα1c (JN180942.1), *Carassius auratus* Nkaα3 (BAB60722.1), *Dicentrarchus labrax* Nkaα1a (AKQ12834.1), *D. labrax* Nkaα1b (AKQ12835.1), *Electrophorus electricus* Nkaα3 (AJR20273), *E. electricus* Nkaα2 (AJR20272), *E. electricus* Nkaα1c1 (AJR20270), *E. electricus* Nkaα1c2 (AJR20271), *Fundulus heteroclitus* Nkaα1 (AAL18002.1), *F. heteroclitus* Nkaα2 (AAL18003.1), *Oncorhynchus mykiss* Nkaα1a (NP_001117933.1), *O. mykiss* Nkaα1b (NP_001117932.1), *O. mykiss* Nkaα1c, (NP_001117931.1), *O. mykiss* Nkaα2 (NP_001117930.1), *O. mykiss* Nkaα3 (NP_001118102.1), *O. masou* Nkaα1a (BAJ13363.1), *O. masou* Nkaα1b (BAJ13362.1), *Oreochromis mossambicus* Nkaα3 (AAD11455.2), *Periophthalmodon schlosseri* Nkaα1 (AGR87393.1), *P. schlosseri* Nkaα3 (AGR87394.1), and *Trematomus bernacchii* Nkaα3 (AAY30258.1).

A phenogramic analysis revealed that three isoforms of Nkaα1 were present in *H. fossilis* as they were grouped together with Nkaα1 isoforms of other teleosts (Figure 4). As one isoform of Nkaα1 was highly similar to the Nkaα1c1 and another was highly similar to the Nkaα1c2 of the electric eel, *Electrophorus electricus*, they were identified as Nkaα1c1 and Nkaα1c2, respectively (Figure 4). In addition, one isoform of Nkaα1 was clustered closely with Nkaα1b of *A. testudineus*, thus it was identified as Nkaα1b. The other two Nkaα isoforms were identified as Nkaα2 and Nkaα3 as they were grouped under the same clade as Nkaα2 of *E. electricus*, *Oncorhynchus mykiss*, and *Fundulus heteroclitus* and Nkaα3 of *E. electricus*, *Carassius auratus*, *O. mykiss*, *Oreochromis mossambicus*, *and P. schlosseri*, respectively (Figure 4).

**FIGURE 5:**
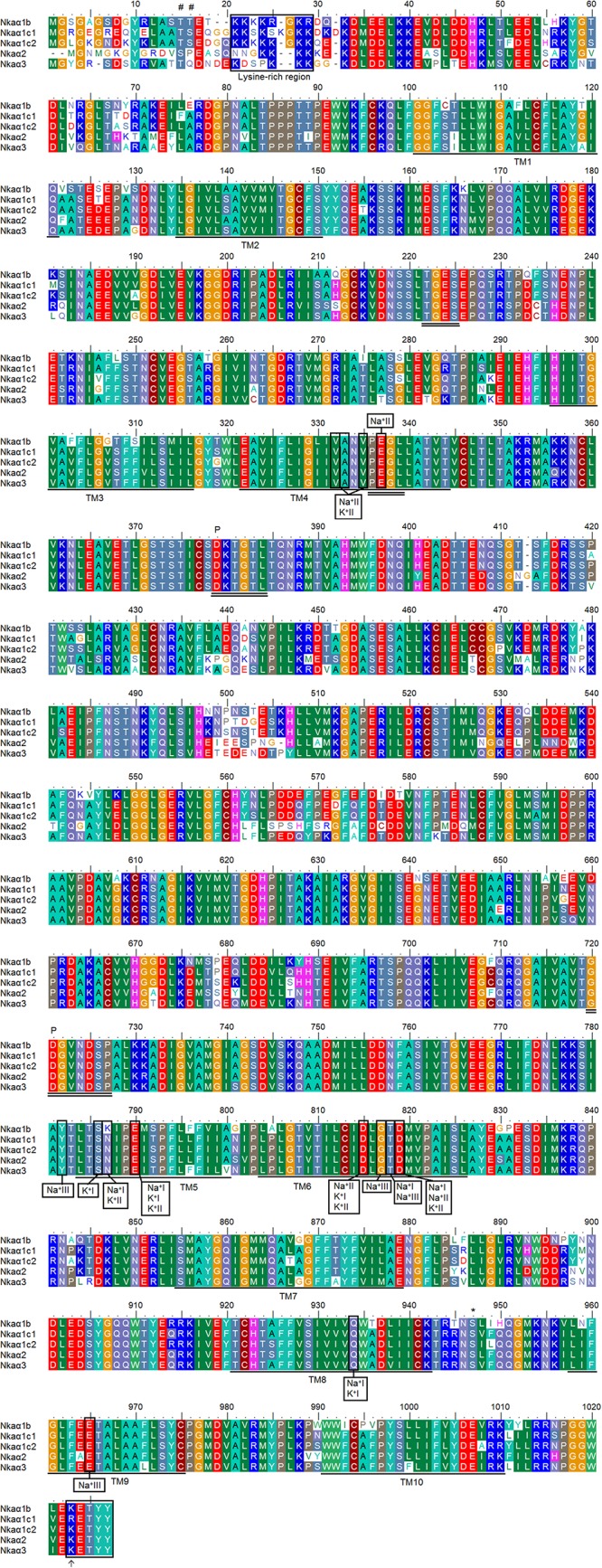
A multiple alignment of the deduced amino acid sequences of the five Na^+^/K^+^-ATPase α (Nkaα) isoforms obtained from the gills of *Heteropneustes fossilis*. Identical or similar amino acid residues are indicated by shaded residues. The 10 predicted transmembrane regions (TM1–TM10) of Nkaα1b, Nkaα1c1, Nkaα1c2, Nkaα2, and Nkaα3 are underlined. Vertical boxes represent coordinating residues for Na^+^ or K^+^ binding. The conserved regions containing the TGES, PEGL, DKTGT, and GDGVNDSP sequence motifs are double underlined and the phosphorylation sites are indicated by a “P.” The asterisk and hash marks denote the amino acid residues phosphorylated by protein kinase A and protein kinase C, respectively. The lysine-rich region is indicated with a box. The KETYY motif is indicated with a box and an arrow indicates the amino acid residue that replaces arginine. The transmembrane domains were predicted using MEMSAT3 and MEMSAT-SVM provided by PSIPRED protein structure prediction server.

The deduced amino acid sequences of all five Nkaα isoforms from the gills of *H. fossilis* had 10 predicted transmembrane domains (TM1-TM10). An alignment of these five amino acid sequences revealed the presence of some highly conserved regions of Nkaα. These include: (1) the threonine-glycine-glutamic acid-serine (TGES) motif that is critical for the catalytic cycle; (2) the proline-glutamic acid-glycine-leucine (PEGL) signature motif in TM4 that is needed for ion binding and transport together with TM5 and TM6; (3) the aspartic acid-lysine-threonine-glycine-threonine (DKTGT) motif that is important for phosphorylation; and (4) the glycine-aspartic acid-glycine-valine-asparagine-aspartic acid-serine-proline (GDGVNDSP) motif that is associated with the phosphorylation site and stabilization of phosphoenzyme intermediates. A highly conserved lysine-rich region that serves as the ion-selective gate important for cation binding and occlusion (Shull et al., 1986) was also present in all five Nkaα isoforms of *H. fossilis*, with Nkaα1c2 containing the greatest number of lysine residues followed by Nkaα1c1, Nkaα1b, Nkaα2, and Nkaα3 (Figure 5).

**FIGURE 6:**
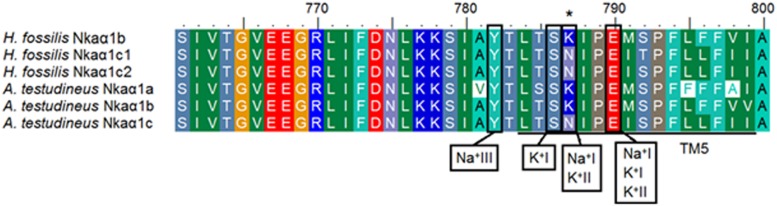
Analysis of Na^+^/K^+^ binding site of the three isoforms of Na^+^/K^+^-ATPase α1 (Nkaα1) from the gills of *Heteropneustes fossilis*. A multiple amino acid sequence alignment of a region of Nkaα1b, Nkaα1c1, and Nkaα1c2 from the gills of *H. fossilis*, with Nkaα1a (GenBank: AFK29492.1), Nkaα1b (GenBank: AFK29493.1), and Nkaα1c (GenBank: AFK29494.1) from the gills of *Anabas testudineus*. Identical or similar amino acid residues are indicated by shaded residues. A vertical box represents the coordinating residue for Na^+^ or K^+^ binding. An asterisk indicates the amino acid residue that is similar to Nkaα1c but different from Nkaα1a and Nkaα1b.

Three Na^+^ (denoted as Na^+^I, Na^+^II, and Na^+^III) and two K^+^ (denoted as K^+^I and K^+^II) binding sites are known to be present in TM4, TM5, and TM6 of NKAα (Ogawa and Toyoshima, 2002). These binding sites were fully conserved in all five Nkaα isoforms of *H. fossilis*, except that asparagine (N) was replaced by lysine (K) at residue 787 in TM5 of Nkaα1b (Figure 5). When the Nkaα1 isoforms (Nkaα1b, Nkaα1c1, and Nkaα1c2) from the gills of *H. fossilis* were aligned and compared with those (Nkaα1a, Nkaα1b, and Nkaα1c) of *A. testudineus* at amino residues 761–800, the amino acid residue (asparagine) constituting the K^+^ binding sites of Nkaα1c1 and Nkaα1c2 of *H. fossilis* was identical to that of Nkaα1c of *A. testudineus* (Figure 6).

### Effects of Exposure to 30 mmol l^–1^ NH_4_Cl on the Transcript Levels of *nka*α*-subunit* Isoforms in the Gills

**FIGURE 7:**
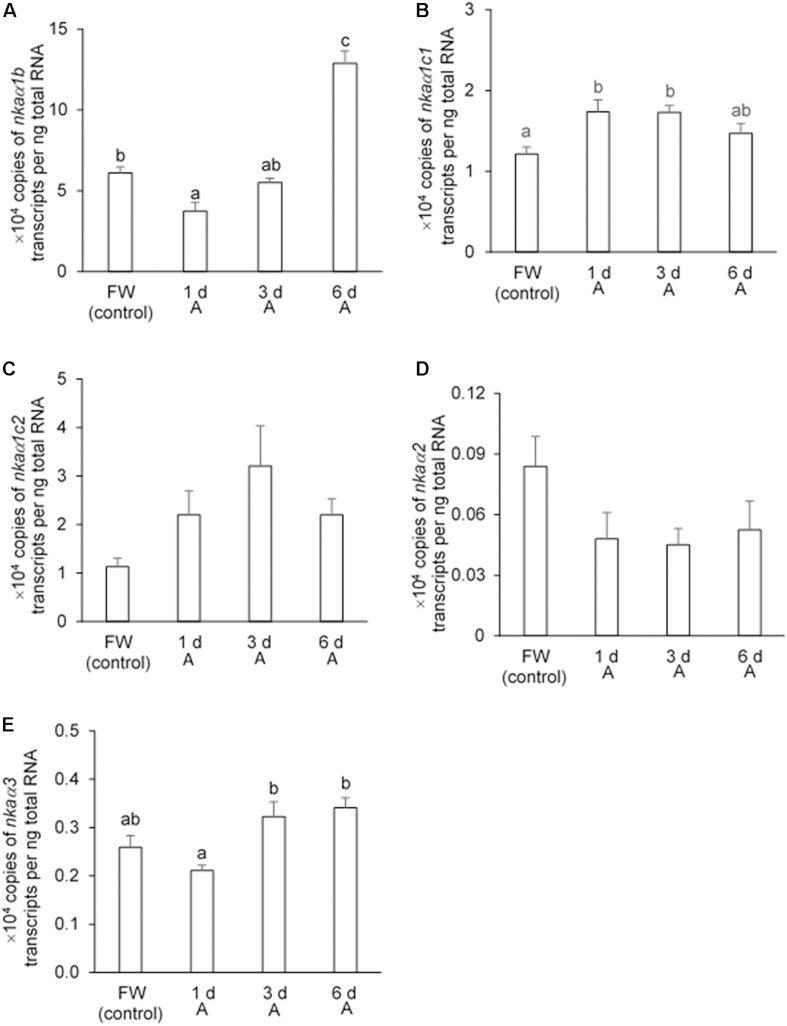
Effects of exposure to 30 mmol l^–1^ NH_4_Cl on the transcript levels of *Na^+^/K^+^-ATPase α-subunit* (*nkaα)* isoforms in the gills of *Heteropneustes fossilis*. Absolute quantification (copies of transcript per ng of total RNA) of **(A)** *nka*α*1b*, **(B)** *nka*α*1c1*, **(C)** *nka*α*1c2*, **(D)** *nka*α*2*, and **(E)** *nka*α*3* in the gills of *H. fossilis* kept in fresh water (FW; control) or exposed to 30 mmol l^–1^ NH_4_Cl (A) for 1, 3, or 6 days (d). Values are mean + SEM (*N* = 5). Means not sharing the same letter are significantly different (*P* < 0.05).

In the gills of *H. fossilis* kept in fresh water (control), the transcript level of *nka*α*1b* was the highest (∼60,000 copies per ng of total RNA; Figure 7A), followed by *nka*α*1c1* (∼12,000 copies per ng of total RNA; Figure 7B), *nka*α*1c2* (∼11,000 copies per ng of total RNA; Figure 7C), *nka*α*3* (∼2600 copies per ng of total RNA; Figure 7E), and *nka*α*2* (∼800 copies per ng of total RNA; Figure 7D). After 1 day of exposure to 30 mmol l^–1^ NH_4_Cl, the transcript level of *nka*α*1b* decreased by 38.5%, but this change was transient and the transcript returned to a level comparable to that of the control after 3 days of ammonia exposure (Figure 7A). Interestingly, exposure of *H. fossilis* to 30 mmol l^–1^ NH_4_Cl for 6 days resulted in ∼2.1-fold increase in the transcript level of *nka*α*1b* in its gills, as compared with that of the freshwater control (Figure 7A). There was also a significant increase (∼1.4-fold) in the transcript level of *nka*α*1c1* after 1 or 3 days of exposure to ammonia (Figure 7B). On the other hand, exposure to 30 mmol l^–1^ NH_4_Cl had no significant effects on the transcript level of *nka*α*1c2* in the gills of *H. fossilis* (Figure 7C). Exposure to ammonia also did not lead to any significant changes in the transcript levels of *nka*α*2* (Figure 7D) and *nka*α*3* (Figure 7E) in the gills of *H. fossilis*.

### Effects of Exposure to 30 mmol l^–1^ NH_4_Cl on the Protein Abundances of Nkaα Isoforms in the Gills

**FIGURE 8:**
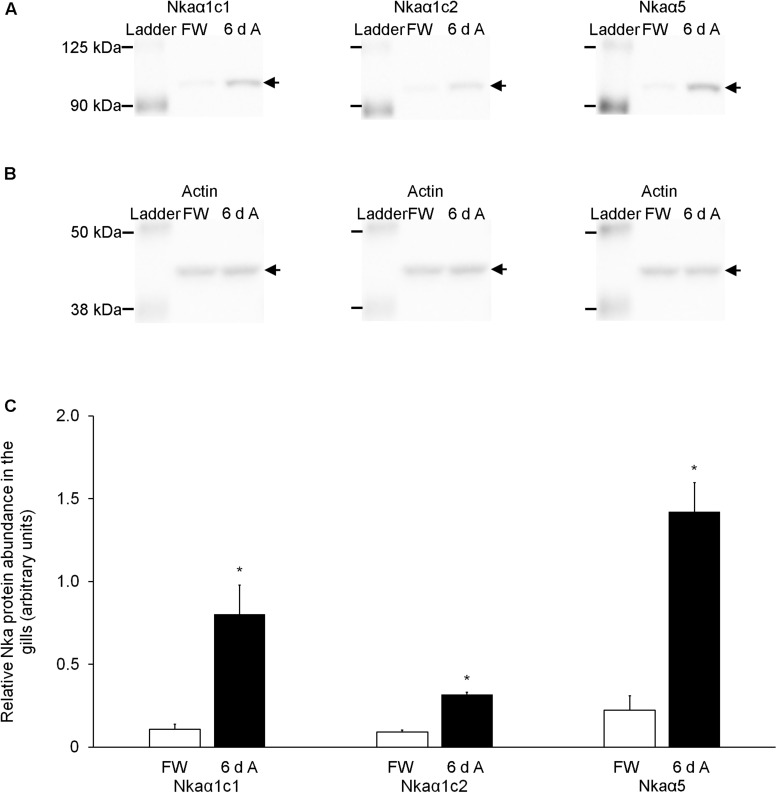
Effects of exposure to 30 mmol l^–1^ NH_4_Cl on the protein abundance of Na^+^/K^+^-ATPase α-subunit (Nkaα) isoforms in the gills of *Heteropneustes fossilis.* Protein abundances of Nkaα1c1, Nkaα1c2, and total Nkaα (as reflected by immunostaining with the α5 comprehensive anti-Nkaα antibody) in the gills of *H. fossilis* kept in fresh water (control; FW) or exposed to 30 mmol l^–1^ NH_4_Cl for 6 days (6 d A). **(A)** An example of the immunoblot of Nkaα1c1, Nkaα1c2, and total Nkaα. **(B)** An example of the immunoblot of actin. **(C)** The protein abundance of the various Nka bands normalized with respect to that of actin. Results represent mean + SEM (*N* = 4). *Significance difference between the NH_4_Cl-exposed group (6 d A; closed bar) and the corresponding freshwater group (FW; open bar) (*P* < 0.05).

The protein abundances of Nkaα1c1 and Nkaα1c2 increased significantly (∼7.4-fold and ∼3.5-fold, respectively) in the gills of fish exposed to 30 mmol l^–1^ NH_4_Cl for 6 days (Figure 8). Overall, there was a significant increase in the protein abundance (∼6.4-fold) of total NKAα (as reflected by immunostaining with the α5 anti-NKAα antibody) in the gills of these experimental fishes as compared with the controls (Figure 8). The peptide competition assay conducted for the anti-Nkaα1c1 and anti-Nkaα1c2 antibodies confirmed the identity of the band of interest obtained for the western blotting results of both Nkaα1c1 and Nkaα1c2 (Supplementary Figure S1).

## Discussion

A ureotelic animal may or may not be able to synthesize urea through the OUC (ureogenesis), because urea can also be produced through the degradation of arginine (argininolysis) or uric acid (uricolysis) (Campbell, 1991; Anderson, 2001). Even if a fish can conduct ureogenesis, it is imperative to examine whether increased urea synthesis is the “major strategy” employed by the fish to detoxify the accumulating ammonia under adverse conditions, and whether the fish also employs other strategies to ameliorate ammonia toxicity. To date, there are very few ureotelic teleosts, and most adult tropical teleosts do not rely on ureogenesis as a major strategy to detoxify ammonia produced internally or infiltrated from the external environment (see Ip et al., 2001a, 2004a, 2004b; Chew et al., 2006; Ip and Chew, 2010, 2018; Chew and Ip, 2014, 2017 for reviews). Even though Lim et al. (2001) detected a complete OUC in the liver of *P. schlosseri*, the CPS activity present was extremely low. Hence, *P. schlosseri* does not depend on increased urea synthesis to detoxify ammonia when it is in a terrestrial environment (Ip et al., 1993, 2001b), high ammonia environment (Peng et al., 1998; Randall et al., 1999), or alkaline medium (Chew et al., 2003a). Instead, it resorts to excreting NH_4_^+^ actively (Randall et al., 1999; Chew et al., 2007, 2014, 2015) and increasing H^+^ excretion (Ip et al., 2004c) through its gills to ameliorate ammonia toxicity during ammonia or terrestrial exposure. Here, we report that likewise, *H. fossilis* is non-ureotelic and non-ureogenic, but capable of active ammonia excretion.

### No Increases in Urea Excretion or Accumulation in *H. fossilis* Exposed to Ammonia

Our results confirmed that *H. fossilis* was non-ureotelic in fresh water or during 6 days of exposure to 30 mmol l^–1^ NH_4_Cl. It did not accumulate urea in its liver, muscle, or plasma during ammonia exposure. In fact, the muscle urea concentration decreased to 0.431 μmol urea−N g^–1^ (compared to the control level of 1.17 μmol urea−N g^–1^) after 6 days of exposure to 30 mmol l^–1^ NH_4_Cl. Hence, it can be concluded that ammonia exposure does not induce ureogensis in *H. fossilis*. By contrast, after exposure to 30 mmol l^–1^ NH_4_Cl for 6 days, the muscle ammonia concentration increased from 1.81 to 3.74 μmol ammonia−N g^–1^, indicating that high tissue ammonia tolerance could be an important strategy to deal with ammonia toxicity in *H. fossilis*. Notably, high tissue ammonia tolerance can accommodate high concentrations of ammonia in the plasma, which could lower the environment-to-body NH_3_ and/or NH_4_^+^ gradients and reduce the rate of exogenous ammonia influx. By contrast, detoxifying the exogenous ammonia that has entered the body into urea through the OUC would maintain an inwardly driven NH_3_ gradient and a continuous influx of NH_3_. That could be one of the reasons why ureogenesis is not commonly utilized by adult teleost fishes to defend against environmental ammonia (see reviews by Ip et al., 2001a, 2004a, 2004b; Chew et al., 2006; Ip and Chew, 2010, 2018; Chew and Ip, 2014, 2017).

### No Detectable CPS III or I Activities in the Liver of *H. fossilis*

Based on the radiometric assay employed in this study, *H. fossilis* lacked detectable CPS I and III activities in its liver. Hence, it does not possess a functional hepatic OUC, and is non-ureogenic. The discrepancy between the results obtained in this study and those of Saha and Ratha (1989, 1994) and Saha et al. (1997) could be due to differences in the CPS assay methods employed. The CPS assay adopted by Saha and Ratha (1989, 1994) and Saha et al. (1997) was a colorimetric method involving a coupled enzyme system whereby commercially available ornithine transcarbamoylase was added to the assay medium. The accuracy and sensitivity of such a coupled enzyme system can be affected by the purity and source of the enzymes used. Such a discrepancy of the activities of CPS had also been reported by Ip et al. (2004a) and Tay et al. (2006), who were unable to detect activities of CPS I or III in the livers of the Asian walking catfish, *Clarias batrachus*, and *A. testudineus* using the radiometric method, although the presence of CPS activities in these fishes has been reported by Saha and Ratha (1989) and Saha et al. (1999) based on the colorimetric method. Taken together, our results explain why freshwater *H. fossilis* is ammonotelic, excreting more than 80% of its nitrogenous wastes (ammonia−N + urea−N) as ammonia−N. *A priori*, the small quantity of urea excreted by *H. fossilis* could originate from argininolysis and/or uricolysis.

### Active Excretion of Ammonia in *H. fossilis* Against 8 mmol l^–1^ NH_4_Cl

Active excretion of ammonia is the most effective way for fishes to defend against ammonia toxicity during terrestrial or ammonia exposure, as it would result in low internal ammonia concentrations and prevent ammonia intoxication in the brain (Peng et al., 1998; Ip et al., 2005). We report for the first time that *H. fossilis* could excrete ammonia continuously in spite of being immersed in water containing 8 mmol l^–1^ NH_4_Cl. The external water containing 8 mmol l^–1^ NH_4_Cl (pH 7.0) would have a NH_3_ concentration of 0.036 mmol l^–1^ and NH_4_^+^ concentration of 7.96 mmol l^–1^ calculated according to the Henderson–Hasselbalch equation. In comparison, the NH_3_ and NH_4_^+^ concentrations in the blood of *H. fossilis* were calculated to be 0.0028 and 0.239 mmol l^–1^, respectively, based on a blood pH of 7.41 (Ip and Chew, unpublished data) and a blood ammonia concentration of 0.242 mmol l^–1^ (Figure 2A). Therefore, it can be concluded that *H. fossilis* has the ability to actively excrete ammonia (possibly NH_4_^+^) despite being confronted by inwardly-directed NH_3_ and NH_4_^+^ gradients. To date, there are three tropical air-breathing teleosts, *P. schlosseri* (Peng et al., 1998; Randall et al., 1999; Ip et al., 2005; Chew et al., 2007), *A. testudineus* (Tay et al., 2006), and *C. gariepinus* (Ip et al., 2004d), which can actively excrete NH_4_^+^. These three fishes can survive for at least 6 days in 75–150 mmol l^–1^ NH_4_Cl, which is far greater than the ammonia concentration of less than 10 mmol l^–1^ NH_4_Cl tolerated by other fishes in the literature. It has been reported that active NH_4_^+^ excretion in the gills of both *A. testudineus* (Ip et al., 2012b) and *P. schlosseri* (Chew et al., 2014) involves NKA as part of the NH_4_^+^ transport mechanism.

### Molecular Characterization of Nkaα Isoforms From the Gills of *H. fossilis*

NKA/Nka is a universal membrane bound enzyme that serves to actively transport three Na^+^ out of the cell and two K^+^ into the cell, supported by energy from ATP hydrolysis (Sweader, 1989). The functioning of this pump is critical for normal cellular functions, such as maintaining the osmotic balance and membrane potential across cells, and providing the driving force for transport of molecules such as glucose and amino acids (Therien and Bolstein, 2000). NKA comprises two major subunits, α and β, which function as a αβ heterodimer. The α-subunit is a large (110–120 kDa) protein with all the functional sites that serve for the catalytic functioning of the enzyme (Blanco and Mercer, 1998). We have identified five different Nkaα isoforms (Nkaα1b, Nkaα1c1, Nkaα1c2, Nkaα2, and Nkaα3) from the gills of *H. fossilis*. Since Nkaα is responsible for the catalytic and transport functions of Nka, various Nkaα isoforms expressed by a fish may exhibit specific differences in their affinities to Na^+^ and K^+^ (see reviews by Hwang and Lee, 2007; Hwang et al., 2011). These differential affinities of branchial Nkaα isoforms toward Na^+^ and/or K^+^ are important in euryhaline teleosts, such as the freshwater- and seawater-acclimated tilapia, pufferfish, and milkfish for both hypoosmotic and hyperosmotic osmoregulation, respectively (Lin and Lee, 2005).

Based on comparison with the human NKAα (Ogawa and Toyoshima, 2002), amino acid residues involved in binding of cations in all five Nkaα isoforms of *H. fossilis* were conserved except for amino acid residue 787 of Nkaα1b where asparagine was substituted by lysine in the Na^+^ binding site I and K^+^ binding site II (Figure 6). Mutational studies have demonstrated the importance of asparagine for both Na^+^ and K^+^ binding (Pedersen et al., 1998). Substitution of asparagine with alanine severely reduced Na^+^ and K^+^ binding, while substitution with glutamine only reduced Na^+^ binding. Hence, the Na^+^ and K^+^ affinities of Nkaα1b could be different from those of Nkaα1c1 and Nkaα1c2 in *H. fossilis* and NKA/Nka of other animal species. According to Jorgensen (2008), the presence of a lysine residue instead of asparagine residue at amino acid position 787 in the Nkaα1b of the rainbow trout and Atlantic salmon would reduce the Na^+^:ATP ratio and the work done in one Na^+^/K^+^ pump cycle of active Na^+^ transport. This would render Nkaα1b energetically suitable for Na^+^ transport against extreme electrochemical gradients during iono-regulation in fresh water. It is therefore logical to deduce that Nkaα1b in the gills of *H. fossilis* could be involved in ion uptake in fresh water.

The amino acid residues constituting the K^+^ binding sites of Nkaα1c1 and Nkaα1c2 from *H. fossilis* are identical to those of Nkaα1c, but different from those of Nkaα1a and Nkaα1b, from the gills of *A. testudineus* (Figure 6). Ip et al. (2012b) identified the Nkaα1a and Nkaα1b from the gills of *A. testudineus* as the freshwater isoform and the seawater isoform, respectively, and demonstrated the possible involvement of Nkaα1c in active ammonia excretion when the fish is confronted with environmental ammonia. They (Ip et al., 2012b) concluded that there was a reduction in the effectiveness of NH_4_^+^ to substitute for K^+^ in the activation of Nka during ammonia exposure, and the up-regulation of Nkaα1c expression functions to remove excess Na^+^ from, and to transport K^+^ in preference to NH_4_^+^ into, the ionocytes so as to maintain intracellular Na^+^/K^+^ homeostasis. High similarities in K^+^ binding sites between Nkaα1c1/Nkaα1c2 of *H. fossilis* and Nkaα1c of *A. testudineus* denote that they may exhibit high substrate specificity for K^+^ and could participate in active ammonia excretion during ammonia exposure.

### Ammonia Exposure Increases the Protein Abundances of Nkaα1c Isoforms and Total Nkaα in the Gills of *H. fossilis*

It is apparent that the expression of Nkaα isoforms in the gills of *H. fossilis* could be regulated at transcriptional and translational levels. Although molecular characterization revealed that Nkaα1b was unlikely to play a part in the multi-component mechanism of active ammonia excretion in *H. fossilis*, its transcript level increased significantly on day 6 of exposure to 30 mmol l^–1^ NH_4_Cl. However, our attempt to develop a specific anti-NKAα1b to determine the protein abundance of NKAα1b in the gills of the control and experimental fish was unsuccessful. Hence, it remains inconclusive whether NKAα1b is a part of the mechanism involved in active ammonia excretion in *H. fossilis*. Importantly, there were significant increases in the transcript levels and/or protein abundances of Nkaα1c1, Nkaα1c2, and total Nkaα in the gills of *H. fossilis* exposed to ammonia. These results indirectly support the proposition that *H. fossilis* can actively excrete ammonia, with reference to our knowledge on *A. testudineus* and *P. schlosseri*. They also corroborate the deduction based on molecular characterization that Nkaα1c1 and Nkaα1c2 could be essential parts of the mechanism that partake in active ammonia excretion in *H. fossilis*. In the case of *A. testudineus*, active ammonia excretion through its gills involves a type of Nka-immunoreactive ionocyte which co-expresses a basolateral Na^+^:K^+^:2Cl^–^ cotransporter 1a (NKCC1a) and an apical cystic fibrosis transmembrane conductance regulator Cl^–^ channel (Ip et al., 2012b, c; Loong et al., 2012). NH_4_^+^ can enter this ionocyte through the basolateral Nkcc1a before being actively transported across the apical membrane. The operation of Nkcc1a in the gills of *A. testudineus* during active ammonia excretion would lead to an increase in the intracellular Na^+^ concentration of the ionocyte, and therefore an up-regulation of Nka activity would be necessary to remove the excess Na^+^. Specifically, exposure to environmental ammonia leads to significant increases in the mRNA expression of *nka*α*1c*, the overall Nka protein abundance, the Nka activity, and the *K*_m_ for NH_4_^+^ relative to K^+^ in the gills of *A. testudineus*. Hence, the up-regulation of *nka*α*1c* expression in the gills of ammonia-exposed *A. testudineus* may serve to remove excess Na^+^ from, and to transport K^+^ in preference to NH_4_^+^ into, the cell in order to maintain intracellular Na^+^ and K^+^ homeostasis (Ip et al., 2012b). Similarly, the increased protein abundances of Nkaα1c1 and Nkaα1c2 in the gills of *H. fossilis* exposed to ammonia indicate the possible involvement of NKCC and the needs to maintain both the intracellular Na^+^ and K^+^ concentrations in the branchial ionocytes involved in active ammonia excretion. Experiments are being performed in the authors’ laboratories to verify the validity of this proposition.

## Conclusion

Our results confirmed that *H. fossilis* is ammonotelic and does not switch to ureotelism when exposed to environmental ammonia. In fact, it does not possess detectable CPSIII or CPSI activities in its liver, and is therefore non-ureogenic. However, it can actively excrete ammonia during ammonia exposure, which also leads to increases in the protein abundances of Nkaα1c1, Nkaα1c2, and total NKAα in its gills. Hence, it can be concluded that active ammonia excretion through the gills with the indirect aid of branchial NKA, but not increased urea synthesis and excretion, engenders the high environmental ammonia tolerance of *H. fossilis*.

## Data Availability Statement

The datasets generated for this study can be found in the GenBank: *nka*α*1b* (MH427004), *nka*α*1c1* (MH427006), *nka1*α*c2* (MH427005), *nka*α*2* (MH427007), and *nka*α*3* (MH427008).

## Ethics Statement

The animal study was reviewed and approved by the Nanyang Technological University Institutional Animal Care and Use Committee which gave the approval for fish maintenance and experimentation under the protocol ARFSBS/NIE-A-0311.

## Author Contributions

SC and YI designed the experiments and wrote the manuscript. ST, SI, and CP performed the experiments and analyzed the data. SC, SI, and KH participated in animal subjection and sample collection. SC, KH, and YI were involved in the analysis of data and approval of the manuscript.

## Conflict of Interest

The authors declare that the research was conducted in the absence of any commercial or financial relationships that could be construed as a potential conflict of interest.

## Footnotes

1 http://web.expasy.org/translate/

2 http://bioinf.cs.ucl.ac.uk/psipred/

## Supplementary Material

FIGURE S1 Protein abundances of Nkaα1c1 and Nkaα1c2 in the gills of *H. fossilis* exposed to 30 mmol l^–1^ NH_4_Cl for 6 days (6 d A). **(A)** An example of the immunoblot of Nkaα1c1, (left) and Nkaα1c1 pre-incubated with immunizing peptide for the peptide competition assay (PCA; right). **(B)** An example of the immunoblot of Nkaα1c2 (left) and Nkaα1c2 pre-incubated with immunizing peptide for the peptide competition assay (PCA; right).

TABLE S1 Primer sequences used for RACE-PCR and qPCR of *Na^+^/K^+^-ATPase* (*nka*) α*-subunit* isoforms.
