# Supplementary figures and images for: The Non-ureogenic Stinging Catfish, Heteropneustes fossilis, Actively Excretes Ammonia With the Help of Na+/K+-ATPase When Exposed to Environmental Ammonia
Source: Front Physiol. 2020 Jan 22;10:1615. doi: 10.3389/fphys.2019.01615 (PMC6987325; doi:10.3389/fphys.2019.01615)

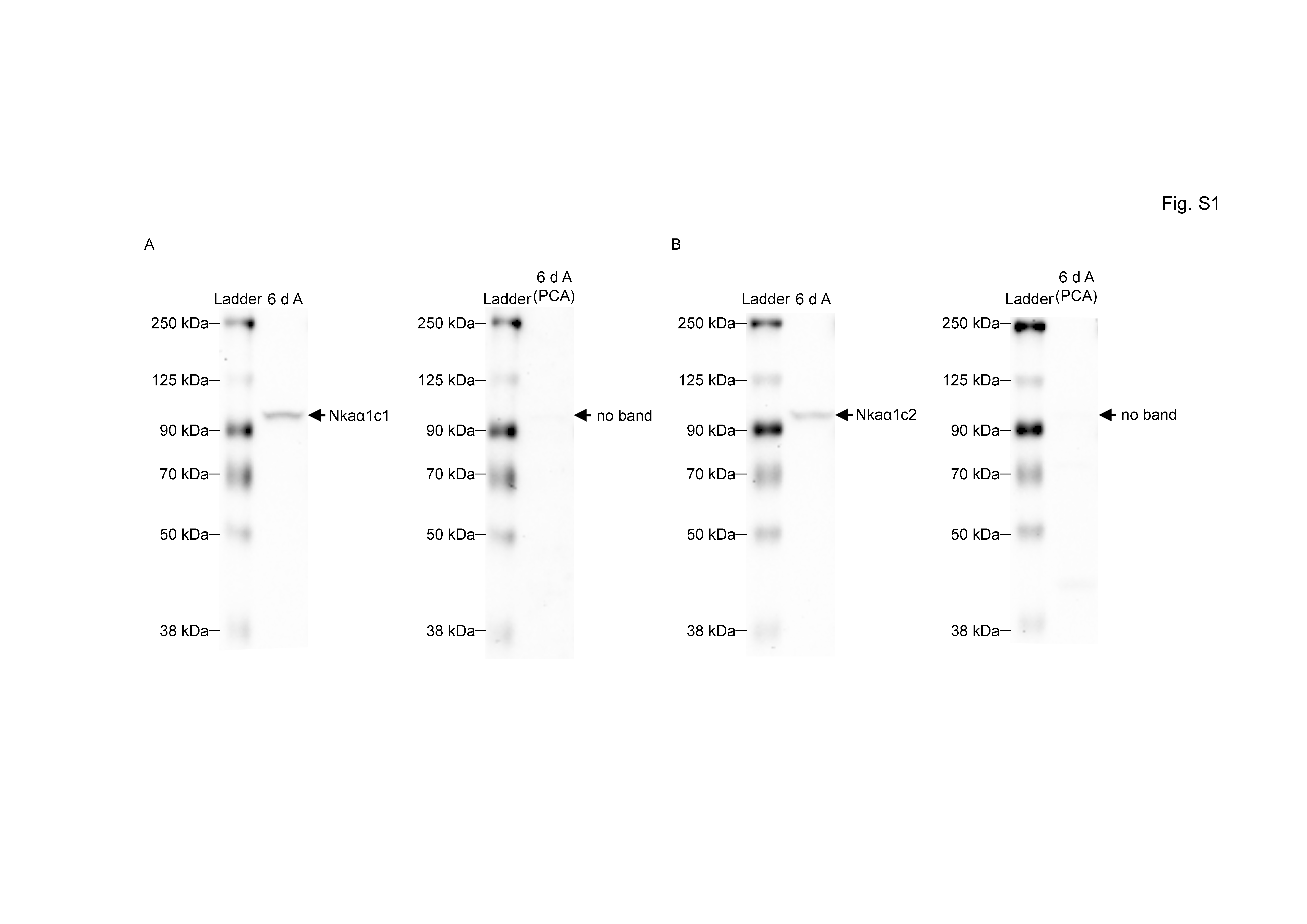

Supplement: FIGURE S1 — Protein abundances of Nkaα1c1 and Nkaα1c2 in the gills of H. fossilis exposed to 30 mmol l–1 NH4Cl for 6 days (6 d A). (A) An example of the immunoblot of Nkaα1c1, (left) and Nkaα1c1 pre-incubated with immunizing peptide for the peptide competition assay (PCA; right). (B) An example of the immunoblot of Nkaα1c2 (left) and Nkaα1c2 pre-incubated with immunizing peptide for the peptide competition assay (PCA; right). [file Image_1.TIF]
