# Supplementary material for: The Non-ureogenic Stinging Catfish, Heteropneustes fossilis, Actively Excretes Ammonia With the Help of Na+/K+-ATPase When Exposed to Environmental Ammonia
Source: Front Physiol. 2020 Jan 22;10:1615. doi: 10.3389/fphys.2019.01615 (PMC6987325; doi:10.3389/fphys.2019.01615)
Supplement: TABLE S1 — Primer sequences used for RACE-PCR and qPCR of Na+/K+-ATPase (nka) α-subunit isoforms. [file Table_1.PDF]

Supplementary Table 1. Primer sequences used for RACE-PCR and qPCR of *Na⁺/K⁺-ATPase* (*nka*) *α-subunit* isoforms

Gene	Primer Type	Sequence (5'–3')
nkaα1b	5'RACE-PCR	TCCTAGAACTCTTTCTCCAAGACCACCCAG
	3'RACE-PCR	CTGGGTGGTCTTGGAGAAAGAGTT
	qPCR	GCATCTTGTCTATGATTCTTGG
		TGACTGTGGCTAGTAATCCTT
nkaα1c1	5'RACE-PCR	CAGTTGACACGCACTCCTATTAAGTCTG
	3'RACE-PCR	TAACCAGATCCACGAAGCCGACACC
	qPCR	AACACTTGCTCGTAATGAAAGG
		AAGACCGCCAAGTTCCAC
nkaα1c2	5'RACE-PCR	CATAATAGTTGAGCATCGATCCAGAATCC
	3'RACE-PCR	TAACCAGATCCACGAAGCCGACACC
	qPCR	TTGTCCATCCACAAGAACC
		CCCAACTCCAAGTAGGCA
nkaα2	5'RACE-PCR	CTGAAACTTGTTGGTTGAGTTGAAGGGAA
	3'RACE-PCR	TTCCCTTCAACTCAACCAACAAGTTTCAG
	qPCR	TTCATTCTTCGTCCTCTCACTC
		CAAAGTCTCTACAGCCTCCA
nkaα3	5'RACE-PCR	CTCTGCTACTTTCTTGTTCTTGTCCCTCAT
	3'RACE-PCR	GAGGGACAAGAACAAGAAAGTAGCAGAGAT
	qPCR	GGACAAGAACAAGAAAGTAGCA
		CATCACCAGCAGATAGGGAG
